# Effects of Active Components of Fuzi and Gancao Compatibility on Bax, Bcl-2, and Caspase-3 in Chronic Heart Failure Rats

**DOI:** 10.1155/2016/7686045

**Published:** 2016-12-08

**Authors:** Liqin Wang, Yu He, Yuyan Zhang, Huifen Zhou, Li Yu, Jiehong Yang, Haitong Wan

**Affiliations:** Zhejiang Chinese Medical University, 548 Binwen Road, Hangzhou, Zhejiang 310053, China

## Abstract

Hypaconitine (HA) and glycyrrhetinic acid (GA) are active components of Fuzi (*Aconitum carmichaelii*) and Gancao (*Glycyrrhiza uralensis *Fisch); they have been used in compatibility for chronic heart failure (CHF) from ancient times. The purpose of the present research was to explore whether apoptosis pathways were related with the protective effects of HA + GA against CHF rats or not. The rats were progressed with transverse-aortic constriction (TAC) operation for 4 weeks to build the CHF state, and then the Digoxin (1 mg/kg), HA (2.07 mg/kg), GA (25 mg/kg), and HA (2.07 mg/kg) + GA (25 mg/kg) were orally administrated to rats for 1 week. The levels of BNP and cTnI in the plasma were decreased in the HA + GA group, and the heart/body weight ratio (H/B) and left ventricular (LV) parameters of transthoracic echocardiography were also declined; moreover, the expressions of Bax, Bcl-2, and caspase-3 were all improved in the HA + GA group than other groups in the immunohistochemistry and western blot methods. In general, the data suggested that Fuzi and Gancao compatibility could protect the CHF rats from apoptosis, which provided a strong evidence for further searching for mechanisms of them.

## 1. Introduction

**Figure 1:**
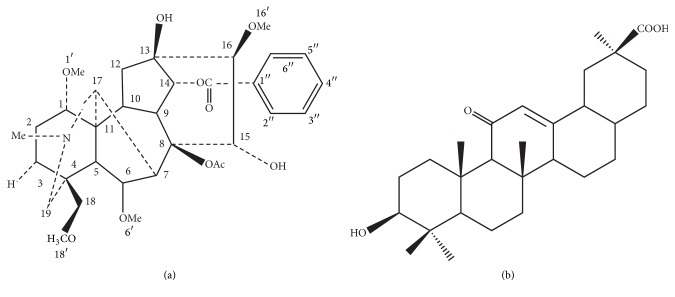
(a) Chemical structure of hypaconitine (HA), molecular formula: C_33_H_45_NO_10_; (b) chemical structure of Glycyrrhetinic acid (GA), molecular formula: C_30_H_46_O_4_.

Fuzi is the processed lateral root of* Aconitum carmichaelii Debeaux*; it has been used in traditional Chinese medicine (TCM) for thousands of years. Fuzi has widespread biological activities, besides the effects on cardiovascular system; there are also including many other systems effects, such as the immune system, the kidney system, and the metabolism system. The common effects reveal as anti-inflammation and analgesic actions, antitumor activity, and antiaging. It has been extensively used for chronic heart failure (CHF), low blood pressure, coronary heart disease, and so on. It is said that 122 chemical constituents have been isolated and identified from Fuzi, among which C_19_-diterpenoid alkaloids and C_20_-diterpenoid alkaloids are the predominant ingredients [1]. Aconite alkaloids have been proved to be primary therapeutic as well as toxic in Fuzi. The aconitines of Fuzi, such as aconitine, hypaconitine (HA), and mesaconitine, were detected through gut sac model and in situ single-pass intestinal perfusion model in the Fuzi-Gancao herb-pair precipitation. The results indicated that 3 diester diterpenoid alkaloids in Fuzi-Gancao could be decomposed in gastrointestinal tract and then absorbed in blood after oral administration, and the permeability of HA appeared best in ileum [2]. The chromatographic fingerprinting of HA was achieved [3], and the structure of HA is shown in Figure 1(a). It has been found that the* T* (max) of HA delayed significantly in DahuangFuzi Decoction group compared with the value of Fuzi group; that is to say, HA has a longer metabolite time in vivo, maybe it has a better effect time with combination herbs than other aconitines [4].

In TCM, Gancao (*Glycyrrhiza uralensis Fisch*) is always used as a concerted application of herbs, and the acid substance is one of its major activity ingredients. Glycyrrhetinic acid (GA) is the metabolite of glycyrrhizinic acid (GL) in vivo [5]; the fingerprinting of GA had been obtained [6], and the structure of GA is shown in Figure 1(b). There have been many researches about GA, such as the metabolic processes caused by mesaconitine-treated rats that could be mitigated by prophylaxis with GA, which meant that pretreatment with GA might reduce the toxicity of mesaconitine in the metabolic level [7]; GA has also been proved to be a typical active component of Shaoyao-Gancao Decoction for pain relief and be the major active constituent of Kushen-Gancao Decoction after oral administration in rat by HPLC-MS/MS [8, 9]. In another study about the comparative pharmacokinetic profiles of GA, GL, and Gancao-Fuzi-Tang after orally taking GL and Gancao-Fuzi-Tang, the results demonstrated an increased effect of GA after oral administration of Gancao-Fuzi-Tang in comparison with GL, which implied the GA was one of the crucial elements in Gancao-Fuzi-Tang [10].

As we all know, TCM consists of many kinds of herbs, and it is a complex system. Every Chinese herb performs one or more specific functions when prescribed alone. Nevertheless, they will play much more complicated effects when used in combination. Because TCM includes lots of chemical components, they influence each other; even sometimes the herb compatibility produces new substances and results in multiply therapeutic effects. These actions are always caused by multicompounds. For example, the Fuzi-Gancao herb-pair generates the reducing toxicities and enhancing effects, may be related on the interaction of acid and base, which brings about some other new chemical compounds [11]. In conclusions, to study the effects of Fuzi-Gancao herb pair, we chose HA and GA on behalf of this herb pair for further investigations.

## 2. Materials and Methods

### 2.1. Material

HA (purity: ≥98%) and GA (purity: ≥98%) were purchased from Baoji Chenguang Biotechnology Co. Ltd. (Xi'an, China); Digoxin pills were obtained from Shanghai sym pharma Co. Ltd. (Shanghai, China); penicillin was bought from REYOUNG Co. Ltd. (Shandong, China); isoflurane was purchased from Shenzhen Huabao Medical Supplies Industry & Trade Development Co., Ltd. (Shenzhen, China); B-type natriuretic peptide (BNP) and cardiac troponin I (cTnI) ELISA kits were bought from Shanghai yuanye biological agent company (Shanghai, China); anti-Bax, anti-Bcl-2, anti-caspase-3, and anti-*β*-actin antibodies were obtained from Santa Cruz Biotechnology Co. (CA, USA); immunohistochemical detection kit was purchased from Beijing Zhongshan Biotechnology Co., Ltd. (Beijing, China) (import packing).

### 2.2. Animal

Healthy male SPF rats (body weight 160–170 g) were provided by Shanghai Slack Laboratory Animal Co. Ltd. (animal license number: SCXK (Shanghai) 2012-0002, animal use certificate number: SYXK (Zhejiang) 2013-184). The rats were watered ad libitum and fed a standard diet. The animals were maintained in the 12 h dark and 12 h light cycle, at the temperature of 20 ± 2°C, and the humidity was 50 ± 2%. All animal experimental protocols were approved by the Animal Care and Use Committee of Zhejiang Chinese Medical University and complied with laboratory animal management and use regulations.

### 2.3. Animal Model

The CHF model was induced by transverse-aortic constriction (TAC) operation, which was modified from deAlmeida's methods [12]. In this study, it was performed after the rats were allowed to acclimate for 1 week. The rats were anesthetized by inhalation of 2.0% isoflurane, and then they were endotracheally intubated with positive-pressure ventilation inputting 100% oxygen gas with a tidal volume of 250 *μ*L at a velocity of 120 breaths per minute by a Small Animal Ventilator (Hallowell, Inc., USA). After opening the chest wall via an upper sternotomy, the aorta was carefully picked out. A self-regulating “L” needle with the external diameter of approximately 0.9 mm was placed on the aorta, on which a 3-0 prolene ligation was placed between the right and left common carotid arteries. The needle was removed cautiously, and the aortic constriction was then created. After the procedure, the wound was closed, respectively, and the animal was allowed to recover from anesthesia with observation. Penicillin was injected intramuscular once a day for 3 days. The rats in Sham group were processed as Model group but without TAC operation.

We chose the TAC operation to create chronic pressure overload responses in the cardiac myocyte, in order to induce CHF, and after 4 weeks' operation, the major situations of rats were in the cardiac hypertrophy period [13].

### 2.4. Experimental Protocol

Rats with TAC operation were fed and watered normally for 4 weeks and then the surviving rats were randomly divided into six groups: Sham group (*n* = 6), Model group (*n* = 6), Digoxin group (*n* = 6), HA group (*n* = 8), GA group (*n* = 7), and HA + GA group (*n* = 7). According to our previous work and professor Chen [14], the dosage of GA orally administered to rats was 25 mg/kg per day. And based on the preliminary tests [15] and the dosage of HA required to kill half of a tested population (LD50 value per day), the dosage of HA was chosen as 2.07 mg/kg per day. Digoxin (1 mg/kg), HA (2.07 mg/kg), GA (25 mg/kg), and HA (2.07 mg/kg) + GA (25 mg/kg) were administered by intragastric once a day for 1 week, and an equal volume of normal saline was fed to the rats in Sham and Model groups. At the end of oral administration, in the HA group two rats died, and the other groups all survived.

### 2.5. Transthoracic Echocardiography

An atraumatic transthoracic echocardiography method [16] was used to evaluate the morphology and function of the heart. Rats were anesthetized by inhalation of 2.0% isoflurane before transthoracic echocardiography by Vevo2100 imaging system (Visual Sonics Inc., Toronto, Canada) with a 30 MHz probe. Stable pictures and measurements were obtained from B-mode and M-mode.

### 2.6. Sample Preparation

After 1 week's treatment, the rats were weighed and anesthetized. The blood was drew from the abdominal aortic, and immediately the plasma was obtained from supernatant of centrifugation of the blood. The heart were gained and weighed. Then, the left ventricular tissues were separated into double parts, one fixed in 4% paraformaldehyde and embedded in paraffin for histological analysis and the other preserved in −80°C for assessing of protein expressions.

### 2.7. Enzyme-Linked Immunosorbent Assay (ELISA)

The plasma samples for ELISA analysis were prepared following the instructions, and levels of BNP and cTnI were detected by ELISA kits, according to the manufacturer's instructions. The optical density (OD) values from the samples were measured with a microplate reader in the 490 nm wavelength.

### 2.8. Immunohistochemistry Staining

Immunohistochemical staining was handled on formalin-fixed, paraffin-embedded 3 *μ*m sections, and the sections were rehydrated. They were incubated with anti-Bax, anti-Bcl-2, and anti-caspase-3 antibody (1 : 200) 60 min at 37°C, rewashed with PBS, and then used horseradish peroxidase coupled goat secondary antibodies incubated 40 min at 37°C. The tissues were then washed by PBS and incubated in Diaminobenzidine (DAB) substrate. The sections were finally counterstained with hematoxylin and xylene before being rewashed in ethanol. Images were collected by DAB light microscope (Leica Microsystems Ltd., Wetzlar, Germany). Integrated optical density (IOD) was obtained to represent the relative amount of positive staining, and 5 fields (magnification 200x) were randomly chosen to analyze. All immunohistochemical results were repeated at least three times, and the representative images were presented.

### 2.9. Western Blot Analysis

The cryopreserved heart tissues were homogenized and then lysed in an ice-cold protein lysis buffer and centrifuged at 14000 rpm for 5 minutes at 4°C to collect proteins [17]. The protein concentrations were determined by BCA methods, using a BCA protein assay kit (Beyotime Biotechnology, Hangzhou, Zhejiang, China). The relevant proteins were separated using 15% SDS-PAGE and transferred to polyvinylidene difluoride (PVDF) membranes, and then they were probed with rabbit anti-rat polyclone anti-Bax, anti-Bcl, and anti-caspase-3 at 4°C overnight. The membranes were then incubated with horseradish peroxidase-conjugated anti-rabbit IgG (1 : 2000) for 2 h at room temperature. The blots were visualized with ECL-Plus reagent (GE Healthcare, Pisca-taway, NJ). In these experiments, the blots were reprobed with an anti-*β*-actin antibody to control the protein loading. Image J (NIH image, Bethesda, MD) was applied to analyze the gel images.

### 2.10. Statistical Analysis

All experimental data are displayed as the mean ± the standard deviation (SD); single factor analysis of variance (ANOVA) was performed using SPSS 19.0 statistical software, followed by Tukey's test. In this study, the value at *P* < 0.05 was considered statistically significant.

## 3. Results

### 3.1. Effects of HA + GA on Left Ventricular (LV) Cardiac Dysfunction and Remodeling

The heart/body weight ratio (H/B) and transthoracic echocardiography results about LV cardiac dysfunction are presented in Table 1. As has been stated above, the rats were in cardiac hypertrophy periods, so the ejection fraction (EF) and fractional shortening (FS) measurements were higher than the CHF standards. But after the treatments, they changed, respectively. Compared with the Sham group, the H/B, EF, and FS were elevated dramatically in the Model group (*P* < 0.01). Compared with the Model group, the H/B, EF, and FS of the HA group had no significant difference (*P* > 0.05), but the EF of GA group and all of the measurements in the Digoxin and HA + GA group were decreased (*P* < 0.05), especially the H/B in Digoxin group and the EF in HA + GA group (*P* < 0.01). The results revealed that HA + GA could protect the CHF rat on regulating LV dysfunctions, particularly on the EF.

### 3.2. Effects of HA + GA on LV Cardiac Remodeling

**Figure 2:**
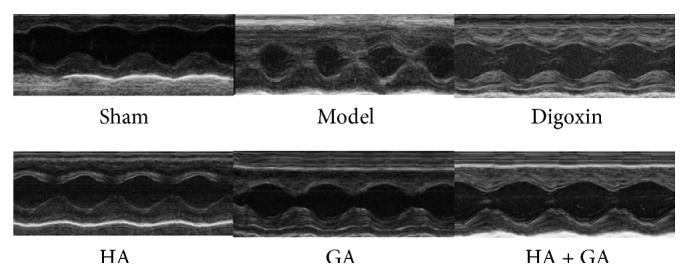
Typical transthoracic echocardiography images from effects of HA + GA on CHF rats. HA: hypaconitine; GA: glycyrrhetinic acid. Sham group (*n* = 6), Model group (*n* = 6), Digoxin group (*n* = 6), HA group (*n* = 6), GA group (*n* = 7), and HA + GA group (*n* = 7).

The results of LV cardiac remodeling in LV end-diastolic dimension (LVIDd), LV end-systolic dimension (LVIDs), the LV end-diastolic posterior wall (LVPWd), and the LV end-systolic posterior wall (LVPWs) were showed in Figure 2 and Table 2. Comparing to the Sham group, the values were all ascended in the Model group (*P* < 0.01). When compared with the Model group, in the measurement of LVIDd, except the HA group (*P* > 0.05), the other groups all declined distinctly (*P* < 0.01); in the measurement of LVIDs, the Digoxin and HA + GA groups descended apparently (*P* < 0.01), the HA and GA groups had no significant difference (*P* > 0.05); in the measurement of LVPWd, the GA group receded it (*P* < 0.05), and the other groups were all reduced dramatically (*P* < 0.01); in the levels of LVPWs, the HA + GA group was obviously declined (*P* < 0.01), and the Digoxin and GA groups (*P* < 0.05), and the HA group had no significance difference (*P* > 0.05). The results demonstrated that the parameters of LV remodeling were significantly modified by the HA + GA treatment, much better than the HA and GA groups.

### 3.3. Effects of HA + GA on CHF Rats in the Levels of BNP and cTnI

**Figure 3:**
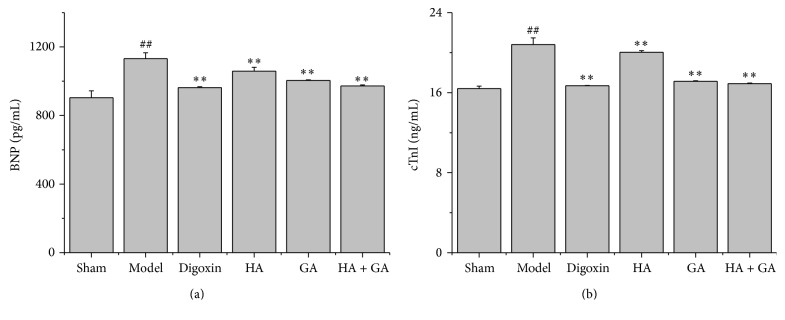
Effects of HA + GA on CHF rats in levels of BNP and cTnI by ELISA kits. (a) The levels of BNP in the plasma; (b) the levels of cTnI in the plasma. HA: hypaconitine; GA: glycyrrhetinic acid. Sham group (*n* = 6), Model group (*n* = 6), Digoxin group (*n* = 6), HA group (*n* = 6), GA group (*n* = 7), and HA + GA group (*n* = 7). All data were presented as mean ± SD. ^##^ *P* < 0.01 versus Sham group; ^*∗∗*^ *P* < 0.01 versus Model group.

Cardiac functions were determined by BNP and cTnI ELISA assays, as revealed in Figures 3(a) and 3(b). In contrast with the Sham group, the Model group extremely elevated the releases of BNP and cTnI levels in the plasma of CHF rats (*P* < 0.01). When comparing with the Model group, the Digoxin, HA, GA, and HA + GA groups all attenuated the levels of BNP and cTnI (*P* < 0.01), and from the amounts of the releases, we could conclude the HA + GA group ameliorated the CHF state better than HA and GA groups.

### 3.4. Effects of HA + GA on Bax, Bcl-2, and Caspase-3 Protein Expressions by Immunohistochemistry

**Figure 4:**
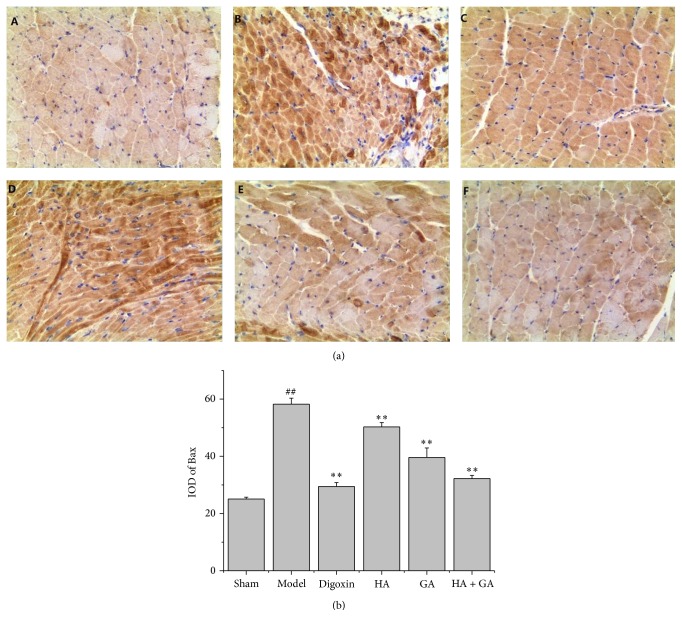
(a) Immunostaining microphotographs of Bax (magnification 200x). (b) Quantitative image analyses of Bax protein were performed based on the IOD of positive immunostaining (brown) in the heart tissues. All data were presented as mean ± SD. ^##^ *P* < 0.01 versus Sham group; ^*∗∗*^ *P* < 0.01 versus Model group. A: Sham group (*n* = 6), B: Model group (*n* = 6), C: Digoxin group (*n* = 6), D: HA group (*n* = 6), E: GA group (*n* = 7), and F: HA + GA group (*n* = 7). HA: hypaconitine; GA: glycyrrhetinic acid.

**Figure 5:**
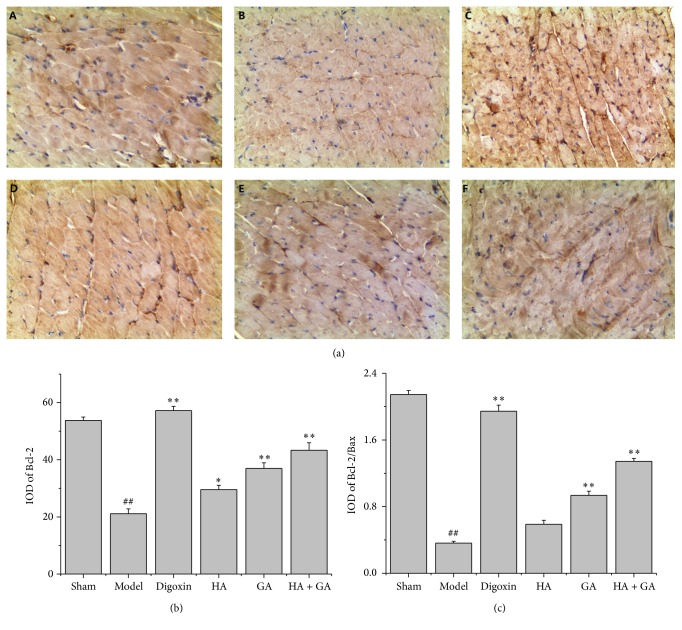
(a) Immunostaining microphotographs of Bcl-2 (magnification 200x). (b) Quantitative image analyses of Bcl-2 protein were performed based on the IOD of positive immunostaining (brown) in the heart tissues. (c) The ratio of Bcl-2/Bax in IOD. All data were presented as mean ± SD. ^##^ *P* < 0.01 versus Sham group; ^*∗*^ *P* < 0.05 and ^*∗∗*^ *P* < 0.01 versus Model group. A: Sham group (*n* = 6), B: Model group (*n* = 6), C: Digoxin group (*n* = 6), D: HA group (*n* = 6), E: GA group (*n* = 7), and F: HA + GA group (*n* = 7). HA: hypaconitine; GA: glycyrrhetinic acid.

**Figure 6:**
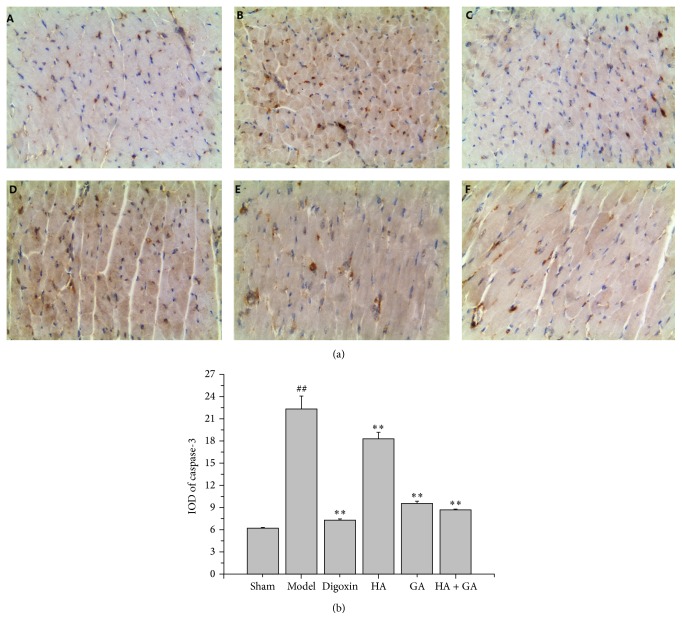
(a) Immunostaining microphotographs of caspase-3 (magnification 200x). (b) Quantitative image analyses of caspase-3 protein were performed based on the IOD of positive immunostaining (brown) in the heart tissues. All data were presented as mean ± SD. ^##^ *P* < 0.01 versus Sham group; ^*∗∗*^ *P* < 0.01 versus Model group. A: Sham group (*n* = 6), B: Model group (*n* = 6), C: Digoxin group (*n* = 6), D: HA group (*n* = 6), E: GA group (*n* = 7), and F: HA + GA group (*n* = 7). HA: hypaconitine; GA: glycyrrhetinic acid.

The immunohistochemistry staining was used to detect the expressions of Bax, Bcl-2, and caspase-3; these proteins were related with apoptosis. In this method, the positive parts were stained to brown as displayed in Figures 4(a), 5(a), and 6(a). The quantitative image analyses were performed based on the IOD of positive immunostaining, as exhibited in Figures 4(b), 5(b), and 6(b); besides, the ratio of Bcl-2/Bax in IOD was showed in Figure 5(c). Comparing to the Sham group, the IOD of Bax and caspase-3 appeared to be uptrend (*P* < 0.01), and the IOD of Bcl-2 and ratio of Bcl-2/Bax performed to be in downtrend (*P* < 0.01). When compared to the Model group, the IOD of Bax and caspase-3 in Digoxin, HA, GA, and HA + GA groups emerged to be dropped (*P* < 0.01), with the HA + GA group holding the least quantity; the IOD of Bcl-2 and ratio of Bcl-2/Bax in Digoxin, GA, and HA + GA groups revealed to be raised dramatically (*P* < 0.01), and the IOD of Bcl-2 in the HA group also increased (*P* < 0.05), but the ratio of Bcl-2/Bax showed no significant difference (*P* > 0.05). We could summarize that the HA + GA group improved the anti-apoptosis effects on the CHF rats.

### 3.5. Effects of HA + GA on Expressions of Bax, Bcl-2, and Caspase-3 by Western Blot Method

**Figure 7:**
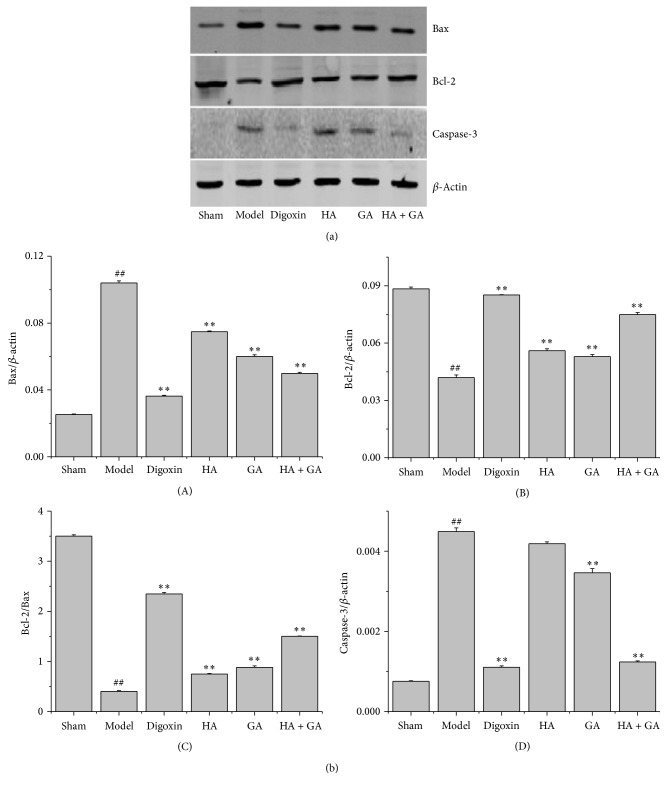
(a) The representative photographs of western blot in expressions of Bax, Bcl-2, and caspase-3 proteins in heart tissues of CHF rats. (b) The quantitative analysis of Bax, Bcl-2, and caspase-3 protein expressions. (A) Expressions of Bax; (B) expressions of Bcl-2; (C) the ratio of Bcl-2/Bax of protein expressions; (D) expressions of caspase-3. HA: hypaconitine; GA: glycyrrhetinic acid. Sham group (*n* = 6), Model group (*n* = 6), Digoxin group (*n* = 6), HA group (*n* = 6), GA group (*n* = 7), and HA + GA group (*n* = 7). All data were presented as mean ± SD. ^##^ *P* < 0.01 versus Sham group; ^*∗∗*^ *P* < 0.01 versus Model group.

The western blot method is a frequently used for further study of protein expressions; in this research, the representative photographs were manifested in Figure 7(a), and the quantitative analysis of protein expressions of Bax, Bcl-2, and caspase-3 was showed in Figure 7(b). By comparison with the Sham group, the Model group elevated the expressions of Bax and caspase-3 proteins (*P* < 0.01); in the meantime, the Model group had lower levels of the expression of Bcl-2 protein and ratio of Bcl-2/Bax (*P* < 0.01). While in contrast with the Model group, except for the expression of caspase-3 in the HA group had no significant difference (*P* > 0.05), all the other groups ameliorated the expressions of Bax and caspase-3 greatly (*P* < 0.01), with the HA + GA group being much less than the HA and GA groups; at the same time, all the groups improved the expression of Bcl-2 protein and ratio of Bcl-2/Bax tremendously (*P* < 0.01), with the HA + GA group in a much higher degree than the HA and GA groups. So we drew the conclusion that the HA + GA could protect the CHF rats from apoptosis better than the HA and GA groups.

## 4. Discussion

CHF has been a worldwide health problem, with a prevalence of over 23 million, rising year by year. Its prevalence greatly increases with advancing age, while the percentage is 0.7% in 45 to 54-year-old people compared with 8.4% in people aged more than 75 years [18]. In clinic, the CHF also has high rates of outpatient visits, hospitalizations, and readmissions. CHF is not a simple single disease, but the final stage of many heart diseases, with a series of clinical syndromes that may have been caused by myocardial hypertrophy, hypertension, ischemic heart disease, and so on [19, 20]. Despite the management and therapy methods advanced, the mortality of CHF still increased along with years. Based on the Framingham Heart Study, 30-day mortality of CHF is approximately 10%, 1-year mortality is about 20–30%, and 5-year mortality is between 45 and 60% [21]. The more effective treatments for CHF patients, the more surviving rates of older patients could be increased [22]. In clinic, almost of the CHF patients take Chinese herbs or patent medicines combined with western medicines, because TCM can both improve the cardiac functions and reduce the side effects of western medicines.

It is generally believed that CHF is based on a complicated pathological mechanism, but without a clear pathogenesis yet [23]. Apoptosis is related with CHF, and great advances have been made in certifying the role of apoptosis in various heart diseases and also in elucidating the mechanisms. Beyond that, the importance of apoptosis in the pathogenesis of CHF and the relationship between cardiac hypertrophic and apoptosis has been investigated [24]. So in this study, we selected the relevant apoptotic proteins, such as Bax, Bcl-2 and caspase-3 to testify the pharmacologic effects and relevant mechanisms of HA + GA on CHF rats.

Cardiac hypertrophy is classified as pathological when associated with cardiac insufficiency. The pathological hypertrophy is induced by lots of factors such as prolonged and abnormal hemodynamic stress, activation of proinflammatory cytokines, and cellular dysfunction, and all these complicated responses will cause cardiac remodeling changes and be the potential process to develop into CHF [25, 26]. Thus, the inhibition of cardiac hypertrophy is a promising therapeutic option for CHF. That is why in this research we give medicines in the cardiac hypertrophy period of CHF. And the results of BNP, cTnI and EF, FS, LVIDd, LVIDs, LVPWd, and LVPWs are consistent with these theories.

From the perspective of TCM, the fundamental problem in heart failure is the deficiency of heart yang and qi, which makes the heart become too weak to transport fluid and move blood and gradually leads to CHF day after day. Fuzi has the functions of warming yang, dispelling cold, removing dampness, and so on. As for yang, the heart yang is the most crucial yang. There have been lots of studies about Fuzi as the major medicine in TCM patent medicines, such as Shenfu decoction (SFD), which is a water extract of Renshen and Fuzi. It has been used for CHF for many years; it can protect myocardial structures, enhance the heart contractility, and even ameliorate the ischemic myocardial metabolism [27]. Though Fuzi has powerful effects in clinic, at the same time, it was confined by its toxic effects. And Gancao has the functions of tonifying qi and detoxifying. In ancient times, TCM doctors used them together, to warming heart yang, tonifying heart qi, and particularly detoxifying. But the mechanisms of their functions are still not so well revealed, so in the present study, we chose the main active components of Fuzi and Gancao to explore the effects and possible related mechanisms on CHF.

As mentioned above, the cardiac hypertrophy is the process of CHF, and we can observe the hypertrophy degree through the analysis of H/B, which is an ordinary measurement index reflecting the cardiac remodeling changes [28]. In this paper, HA + GA group reduced the H/B and showed a better effect than other groups on CHF rats. We acquired that the combination of HA + GA could ameliorate the cardiac remodeling better than the single activity ingredient. And in 2016 ESC guidelines for CHF, it also points out that transthoracic echocardiography is a crucial diagnosis method in CHF [29]. In the meantime, transthoracic echocardiography is also often used in diagnosis of cardiovascular diseases and prognosis of curements. For example, it can measure the thickness of ventricular wall and carotid intima-media, which are risk factors of cardiovascular diseases. And we know that heart failure is a state of most cardiovascular diseases [30, 31]. So in this research, we selected the transthoracic echocardiography for evaluations of cardiac functions. In the results, we confirmed that HA + GA compatibility could descend the parameters of LV remodeling measurements, which are crucial in improving the cardiac functions in rats with CHF.

The correlation between levels in cardiac troponin and degrees in cardiac functions has been well established. In a research the level of cTnI was significantly higher in heart failure patients, and exhibited increasing precise concentrations with rising New York Heart Association classification of heart failure severity, which meant cTnI is an accurate indicator for the diagnosis of heart failure [32]. There are also other studies demonstrated that the release of cTnI is associated with increased 1 year mortality and heart failure related readmission, and the cTnI level can act as an independent predicator, and complete in prognostic utility of CHF patients [33]. The level of plasma BNP is a cost-effective method for evaluating the LV dysfunction. And some studies presented that BNP-guided therapies showed a decrease in death and hospital stay of CHF patients [34]. That means BNP is positively associated with all-cause mortality of CHF, and researchers also found that the standard of BNP was useful in estimating prognosis in stable CHF patients [35]. In our study, we received that the HA + GA group could decrease the levels of cTnI and BNP in plasma of CHF rats, and the cardiac protection was much better than the HA group and GA group, and even nearly equaled to the Digoxin group, which is still one of the major treatments for CHF in 2016 ESC guidelines [29].

As for the relationship between apoptosis and CHF, we used immunohistochemistry assays and western blot methods which belonged to quantitative and qualitative analysis, to detect the levels of apoptotic and antiapoptotic proteins, such as Bax, Bcl-2, and caspase-3, they are on the downstream of apoptotic signal pathway, and they mediate the final morphological and biochemical alterations which are characteristic of apoptosis [24]. The apoptosis process is vital to the survival of the organism, which can cause degenerative diseases if too much and may lead to the onset of proliferative diseases if too little; that is to say, the disturbance in the balance between cell growth and cell death is very crucial in incentives of many diseases, so it is of great significance to explore the protein expressions of apoptosis in diseases [36]. It is well known that Bcl-2 is a protein that inhibits cell apoptosis and leads to cells survival, while Bax is a protein that promotes cell apoptosis and leads to cells death. There were studies revealing that the inhabitation of Bcl-2 and other related antiapoptotic proteins could make proliferation pathologic changes become better [37–39]. Furthermore, a protective effect of ischemic preconditioning was certified to be involved with upregulation of Bcl-2 [40]. Besides, the ratio between antiapoptotic and proapoptotic proteins is considered to be a key index for estimating homeostasis states in survival of cells [41]; for example, the change of Bcl-2/Bax ratio was suggested to promote cell apoptosis in an apoptotic stimulus [42], while other studies demonstrated that the decrease of the Bcl-2/Bax ratio might lead to an increase rate of apoptosis in cardiomyocytes [43].

In the activity of apoptosis pathway, we know that caspase-3 is a fatal factor, and it is one of the major caspases families which are involved in the final execution step of apoptosis process. Caspase-3 is responsible for the cleavage of numerous other apoptosis proteins. In addition, caspase-3 can cause a decrease in the amount of Bcl-2 protein; and in contrast Bcl-2 protein also inhibits caspsae-3 protein [42]. There were studies as well showing that the regulation of caspase-3 could reflect the anti-apoptosis effects in medicines on CHF patients [44]. What is more, the vital role of caspases in apoptosis process makes them a potential target for anti-apoptotic therapies. For example, the over expression of p35 in a pacing-induced CHF model, resulting in the decrease of caspase-3 activity and turned to be related to the improved cardiac functions [45]. In present research, we obtained that the HA + GA group upregulated the expression of Bcl-2 protein and the ratio of Bcl-2/Bax, while it reversely downregulated the expressions of Bax and caspase-3 proteins. That means the combination of HA and GA could protect the cardiomyocytes from apoptosis, and the effects might be associated with apoptotic signal pathways belonging to the mitochondrial pathway, such as PI3K/Akt pathway or others. Although our findings can provide some strategies for modulating cardiac apoptosis, further investigation is required to clarify the exact pathway about the anti-apoptosis effect on CHF rats by Fuzi and Gancao.

## 5. Conclusions

In conclusion, HA + GA could significantly inhibit the development of cardiac hypertrophy, remodeling, and dysfunction in the CHF processing, and the underlying mechanism maybe attributed to the attenuation of cardiac apoptosis. Although the exact mechanism of the effects of HA + GA remains to be further explored, our data point out the direction for the upcoming mechanism research for Fuzi and Gancao compatibility on CHF patients.

## Figures and Tables

**Table 1:** The effects of HA + GA on CHF rats in H/B and LV dysfunction ($\overset{-}{x}\pm s$).

Group	H/B (mg/g)	EF (%)	FS (%)
Sham	0.30 ± 0.01	72.44 ± 0.63	43.35 ± 1.20
Model	0.51 ± 0.02^##^	92.29 ± 0.69^##^	57.08 ± 1.30^##^
Digoxin	0.33 ± 0.01^*∗∗*^	77.40 ± 1.72^*∗*^	48.41 ± 0.91^*∗*^
HA	0.48 ± 0.02	89.59 ± 1.48	54.71 ± 0.17
GA	0.43 ± 0.01	86.69 ± 1.05^*∗*^	52.68 ± 0.58
HA + GA	0.36 ± 0.01^*∗*^	80.56 ± 0.39^*∗∗*^	49.90 ± 0.73^*∗*^

Note: HA: hypaconitine; GA: glycyrrhetinic acid; H/B: the heart/body weight ratio; EF: ejection fraction; FS: fractional shortening. Sham group (*n* = 6), Model group (*n* = 6), Digoxin group (*n* = 6), HA group (*n* = 6), GA group (*n* = 7), and HA + GA group (*n* = 7). All data were presented as mean ± SD. ^##^ *P* < 0.01 versus Sham group; ^*∗*^ *P* < 0.05 and ^*∗∗*^ *P* < 0.01 versus Model group.

**Table 2:** The effects of HA + GA on CHF rats in LV remodeling ($\overset{-}{x}\pm s$).

Group	LVIDd (mm)	LVIDs (mm)	LVPWd (mm)	LVPWs (mm)
Sham	3.75 ± 0.20	2.24 ± 0.17	2.16 ± 0.11	2.53 ± 0.14
Model	8.04 ± 0.28^##^	4.73 ± 0.15^##^	4.56 ± 0.03^##^	4.94 ± 0.21^##^
Digoxin	4.82 ± 0.86^*∗∗*^	2.53 ± 0.08^*∗∗*^	2.38 ± 0.10^*∗∗*^	3.01 ± 0.07^*∗*^
HA	7.54 ± 0.07	4.04 ± 0.02	3.10 ± 0.05^*∗∗*^	4.52 ± 0.11
GA	6.98 ± 0.17^*∗∗*^	3.28 ± 0.27	2.80 ± 0.18^*∗*^	3.78 ± 0.17^*∗*^
HA + GA	6.42 ± 0.15^*∗∗*^	2.75 ± 0.13^*∗∗*^	2.51 ± 0.03^*∗∗*^	3.28 ± 0.19^*∗∗*^

Note: HA: hypaconitine; GA: glycyrrhetinic acid; LV: left ventricular; LVIDd: LV end-diastolic dimension; LVIDs: LV end-systolic dimension; LVPWd: LV end-diastolic posterior wall; LVPWs: LV end-systolic posterior wall. Sham group (*n* = 6), Model group (*n* = 6), Digoxin group (*n* = 6), HA group (*n* = 6), GA group (*n* = 7), and HA + GA group (*n* = 7). All data were presented as mean ± SD. ^##^ *P* < 0.01 versus Sham group; ^*∗*^ *P* < 0.05 and ^*∗∗*^ *P* < 0.01 versus Model group.
